# Triple primary lung cancer: a case report

**DOI:** 10.1186/s12890-022-02111-x

**Published:** 2022-08-19

**Authors:** Hye Sook Choi, Ji-Youn Sung

**Affiliations:** 1 Department of Internal Medicine, Kyung Hee Unversity Medical Center, 23 Kyunghee dae-ro, Dongdaemun-gu, Seoul, 02447 Republic of Korea; 2 Department of Pathology, Kyung Hee University Medical Center, Seoul, Republic of Korea

**Keywords:** Multiple primary lung cancer (MLPC), Synchronous MLPC, Metachronous MLPC, Parking attendant

## Abstract

**Background:**

The risk of developing lung cancer is increased in smokers, patients with chronic obstructive pulmonary disease, individuals exposed to environmental carcinogens, and those with a history of lung cancer. Automobile exhaust fumes containing carcinogens are a risk factor for lung cancer. However, we go through life unaware of the fact that automobile exhaust is the cause of cancer. Especially, in lung cancer patient, it is important to search out pre-existing risk factors and advice to avoid them, and monitor carefully for recurrence after treatment.

**Case presentation:**

This is the first report of a case with triple lung cancers with different histologic types at different sites, observed in a 76-year-old parking attendant. The first adenocarcinoma and the second squamous cell carcinoma were treated with stereotactic radiosurgery because the patient did not want to undergo surgery. Although the patient stopped intermittent smoking after the diagnosis, he continued working as a parking attendant in the parking lot. After 29 months from the first treatment, the patient developed a third new small cell lung cancer; he was being treated with chemoradiation.

**Conclusions:**

New mass after treatment of lung cancer might be a multiple primary lung cancer rather than metastasis. Thus, precision evaluation is important. This paper highlights the risk factors for lung cancer that are easily overlooked but should not be dismissed, and the necessity of discussion with patients for the surveillance after lung cancer treatment. We should look over carefully the environmental carcinogens already exposed, and counsel to avoid pre-existing lung cancer risk factors at work or residence in patients with lung cancer.

## Background

The risk factors for lung cancer include smoking and inhaling exhaust fumes. Primary lung cancer (PLC) increases the risk of secondary lung cancers by four to six times [1, 2]. With increasing exposure to environmental risk factors such as automobile exhaust fumes and advances in computed tomographic (CT) screening and treatment modality of lung cancer, the incidence of multiple primary lung cancers (MPLC) is increasing [2]. Synchronous MPLC is defined as a new cancer if it occurs with the same histology within 2 years after the PLC therapy, or with a different histology at the same time [3]; Metachronous MPLC is defined as a new cancer with the same histology if it occurs after a tumor-free period of 2 years; otherwise, it is considered to have a different histology [3]. Incidence of MPLC is higher in women, people with history of malignant disease, and those with chronic obstructive pulmonary disease (COPD), compared to solitary PLC. Men, smokers, patients with COPD, and those with non-adenocarcinomas have higher incidence of metachronous MPLC. Female sex and not smoking are independent risk factors for synchronous MPLC [4]. It is important to manage the risk factors for MPLC in patients diagnosed with lung cancer. However, patients counselling to avoid the already existing risk factors for lung cancers is not generally conducted in depth. For the first time, we report a case of triple lung cancers with metachronous MPLC in a parking attendant.

### Case presentation

A 76-year-old man was referred for a lung mass in December 2018. He was a smoker (30 pack years with intermittent stops) and parking attendant for 30 years. There was no history of lung cancer in the immediate family of the patient. The patient was administered a dual bronchodilator for COPD.

**Fig. 1:**
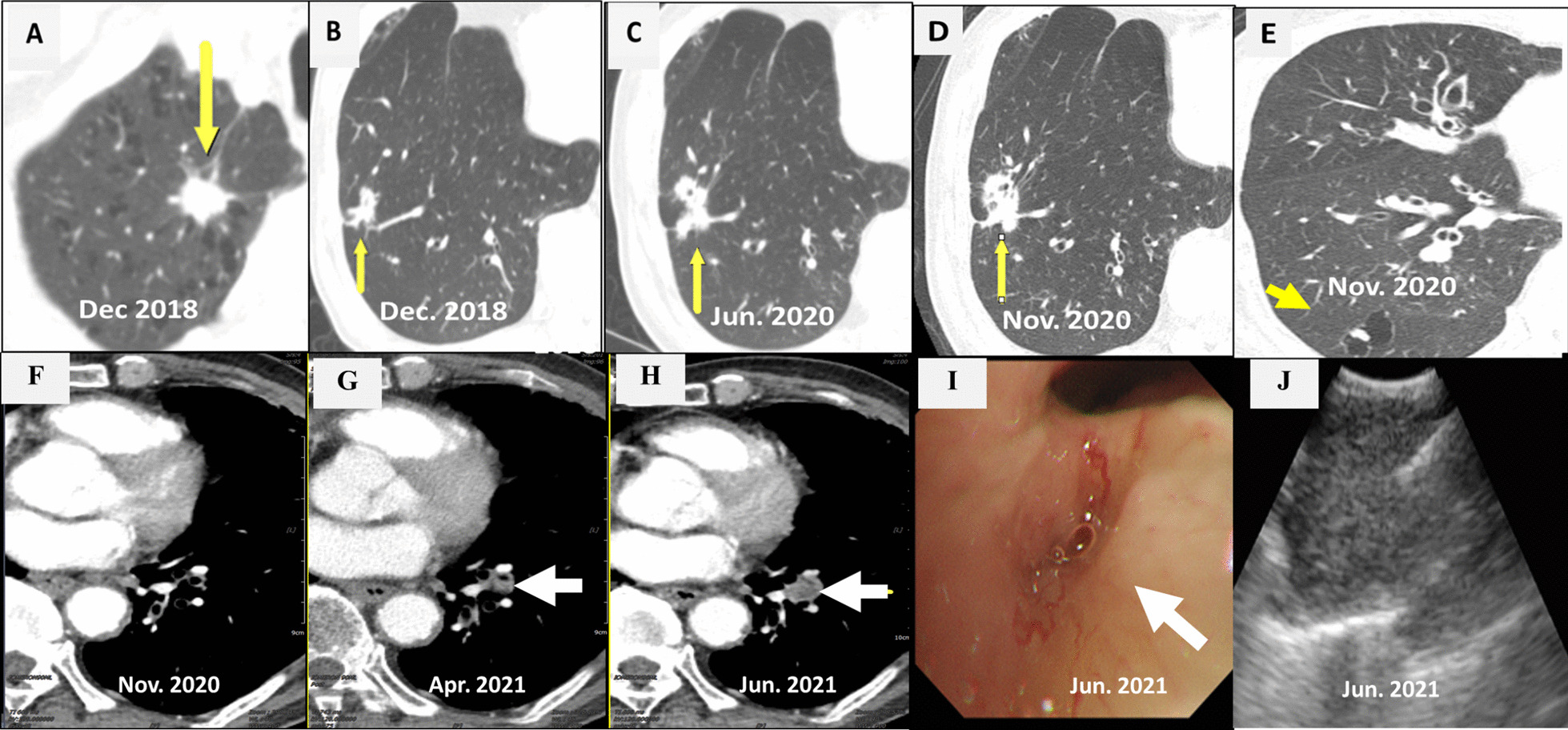
Chest CT scans. **a** A mass on the RUL of the first adenocarcinoma (arrow). **b** A mass on the RLL at the same time of the first cancer diagnosis (arrow). **c** Increased RLL mass six months later (arrow). **d** Further increased RLL mass after five months (arrow). **e** New nodule on the peripheral RLL (arrow). **f–h** Development and increase of the lymph node (arrow). **i** Bronchoscopic finding showing LLL anterobasal segment obstruction (arrow). **j** Lymph node enlargement on the EBUS. CT, computed tomography; RUL, right upper lobe; RLL, right lower lobe; LLL, left lower lobe; EBUS, endobronchial ultrasound

**Fig. 2:**
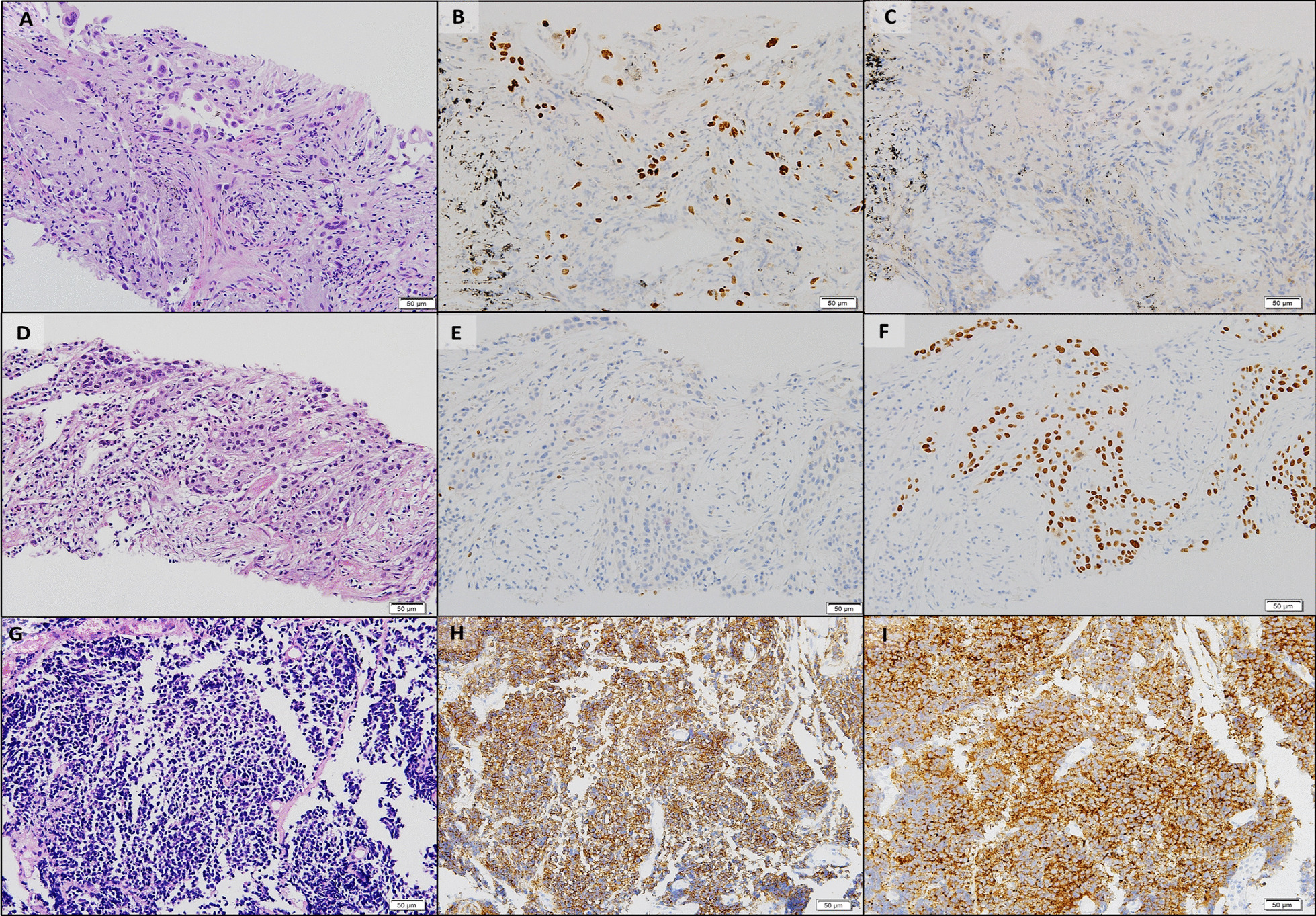
Histopathologic comparisons of the triple lung cancers. **a-c** The first tumor of adenocarcinoma at the right upper lobe. **a** Pleomorphic neoplastic cells with an acinar pattern (hematoxylin and eosin stain, ×200). **b** Immunoreactivity for TTF-1(×200). **c** Negative for P40(×200). **d-f** The second tumor of squamous cell carcinoma at the right lower lobe. **d** Polygonal cells with a solid pattern and no keratinization (hematoxylin and eosin stain, ×200). **e** No immunoreactivity for TTF-1(×200). **f** Strong staining of P40 at tumor cells(×200). **g-i** The third tumor of small cell carcinoma at the left lower lobe. **g** Small cells with scant cytoplasm and lack of nucleoli with a high mitotic activity (hematoxylin and eosin stain, ×200). **h** Positive neuroendocrine markers of CD56(×200). **i** Positive neuroendocrine marker of synaptophysin(×200). Equipment used to obtain images: Olympus BX53 microscope/Olympus objective lens WHN10X/22 UIS2, Olympus DP72 cameras and acquisition software: Olympus CellSens Standard 1.6 software. TTF-1, thyroid transcription factor-1

CT scan showed a 1.4 cm × 1.3 cm mass in the right upper lobe (RUL) (Fig. 1a) and a right lower lobe (RLL) mass-like consolidation (Fig. 1b). Histopathologic examinations of CT-guided-percutaneous needle biopsy (PCNB) of the RUL mass revealed adenocarcinoma (ADC) (Fig. 2a–c) with clinical staging cT1bN0M0 on ultrasonic-guided transbronchial needle biopsy (EBUS-TBNB) and fluorodeoxyglucose F18-positron emission tomography (FDG-PET) scan. RLL mass showed no metabolism on the FDG-PET scan. The FEV_1_ was 56% of the predicted value. We planned a lobectomy for the RUL cancer and a follow-up for the RLL mass. However, the patient refused to undergo surgery and was treated with stereotactic radiosurgery (SRS) on the RUL mass in January 2019. The RLL mass-like consolidation did not show any changes on the follow-up chest CT or FDG-PET scan in November 2019.

In June 2020, the RLL mass-like consolidation was found to have increased on a chest CT scan (Fig. 1c). PCNB of the RLL mass was performed, and histologic examination revealed anthracofibrosis. Five months later, the RLL mass increased further (Fig. 1d), and a new nodule appeared at the periphery of the RLL (Fig. 1e). PCNB was performed again on the same RLL mass (Fig. 1d), and histological examination demonstrated squamous cell carcinoma (SCC) (Fig. 2d–f). There was no metastasis except for hypermetabolism of the new nodule in the RLL periphery (Fig. 1e) on the FDG-PET scans. We could not perform a biopsy for the new peripheral nodule (Fig. 1e) due to cystic changes. We concluded the clinical staging of the RLL SCC as cT3N0M0 on the EBUS-TBNB and PET scan. SRSs were performed separately for the RLL SCC and the new RLL peripheral nodule, respectively in February 2021.

We performed chest CT scan for surveillance of lung cancer. Five months later after 2nd SCC diagnosis, a new nodule emerged at the left lower lobe (LLL) (Fig. 1f, g). Two months after that, the nodule increased further (Fig. 1h). Bronchoscopy showed new total obstruction of the anterobasal segmental bronchus of the LLL (Fig. 1i). Histologic examinations of bronchial biopsy and EBUS-TBNB (Fig. 1j) for LLL lesions demonstrated small cell lung carcinoma (SCLC) (Fig. 2g–i). Clinical staging was limited stage. The patient was treated with chemotherapy (etoposide/carboplatin) and concurrent thoracic radiation.

## Discussion and conclusions

Smoking is a notorious risk factor for lung cancer. The parking attendant was exposed to exhaust fumes, including carcinogens from the fuel. He was using a bronchodilator for COPD. Smoking and COPD are independent risk factors for MPLC [4]. PLC increased the risk of MPLC despite stage IA lung cancer [5, 6]. We suggest that his history of exposure to exhaust fumes in addition to smoking, COPD, and PLC contributed to the metachronous MPLC.

At the time of the first ADC diagnosis on the RUL, we discuss the possibility that the RLL mass was lung cancer, and decided to follow according to the PET-CT scan results with the multidisciplinary approach. Unfortunately, 18 months later, PCNB and histologic findings for the RLL mass showed no cancers. Five months after that (23 months after the first ADC treatment), repeated PCNB on the RLL mass demonstrated SCC. The possibility that an additional abnormality is cancer must be addressed when PLC is diagnosed.

The third SCLC of LLL developed newly 29 months after the first ADC treatment. It was detected after 5 months after the diagnosis of second cancer. Timely CT scan for surveillance is essential for earlier diagnosis of metachronous MPLC in the patients with PLC, which could be improve the outcomes of MPLC. We considered that the first ADC and the second SCC were synchronous MPLC; thus, the third SCLC might be metachronous MPCL. The three different types of MPLC were not a transformation of the PLC after SRSs, but originally developed from three different histologies. Recently, genetic/molecular profiles have begun to be used for differentiation and diagnosis of MPLC [7]. and further investigation is needed.

The primary tumor control rate of SRS is 97.6% in medically inoperable early-stage non-SCLC [8]. Recently, the risk of metachronous MPLC was found to be lower with radiotherapy than non-radiotherapy [6, 8] even though in stage IA lung cancer [5]. The incidence of metachronous MPLC was 0.5% at 1 year and 2.28% at 5 years among solitary PLC survivors with radiotherapy, which was lower compared to the non-radiotherapy group [6]. Based on these findings, it is assumed that the SRSs might not induce metachronous MPLC in our patient.

The question was what could have been responsible for the patient’s triple lung cancers. Unknown susceptible genetic factors, smoking, and exhaust fumes might have contributed to the development of triple lung cancers. Previously reported risk factors [4] such as male sex, smoking, COPD, and nonadenocarcinoma also increased the risk of metachronous MPLC in this patient. He stopped smoking after the first diagnosis of lung cancer, but continued as a parking attendant for 12 h a day. It is well known that harmful effects of smoking persist for years even after smoking cessation. Thus, the main cause of lung cancer in this patient is likely to be smoking. Physicians always counsel their lung cancer patients that smoking is one of the main causes of lung cancer and advise to quit smoking immediately. However, the emphasis on counselling avoidance of other environmental carcinogens that may have a synergistic effect with smoking is often neglected. This patient was exposed to exhaust gas at work for 30 years which is a known occupational carcinogen, and exposure continued even after quitting smoking and diagnosing lung cancer. He had no family history of lung cancer. Unfortunately, his wife was diagnosed with stage IV lung adenocarcinoma lung cancer at August 2021, the time of 3^rd^ SCLC diagnosis of him. He and his wife had worked together in parking lot for several years. We suggest that exhaust fumes might be an additional main risk factor for metachronous MPLC that is easily overlooked in this patient.

Despite stage I lung cancer, careful surveillance for metachronous MPLC is needed, especially in patients with a history of smoking, COPD, PLC, and exposure to environmental carcinogens such as exhaust fumes. Occupation and environment surveys with attentive advice for risk factors of lung cancer are very important, and it is valuable to evaluate concurrent abnormal images in patients with lung cancer. Appropriate CT scan surveillance after PLC therapy can help identify curable MPLC, which might lead to improved overall survival.

## Data Availability

All data generated or analyzed during this study are included in this published article.

## Abbreviations

ADC: Adenocarcinoma
COPD: Chronic obstructive pulmonary disease
CT: Computed tomography
EBUS-TBNB: Ultrasonic-guided transbronchial needle biopsy
FDG-PET: F18-positron emission tomography
LLL: Left lower lobe
PLC: Primary lung cancer
MPLC: Multiple primary lung cancers
PCNB: Percutaneous needle biopsy
RLL: Right lower lobe
RUL: Right upper lobe
SCC: Squamous cell carcinoma
SCLC: Small cell lung carcinoma
SRS: Stereotactic radiosurgery
